# ATG4B-dependent processing of tandem-domain BmLC3 exposes a Tyr136-regulated C-terminal fragment

**DOI:** 10.3389/fcell.2026.1957836

**Published:** 2026-09-29

**Authors:** Won-Dong Shin, Hun-Joo Kim, Sang-Hee Ma, Sang-Won Park, Yong-Woo Jun, Do-Kyung Lee, In-Young Hwang, Deok-Jin Jang, Jin-A Lee

**Affiliations:** 1 Research Institute of Invertebrate Vector, Kyungpook National University, Sangju, Republic of Korea; 2 Department of Vector Entomology, College of Ecology and Environment, Kyungpook National University, Sangju, Republic of Korea; 3 Department of Ecological Science, College of Ecology and Environment, Kyungpook National University, Sangju, Republic of Korea; 4 Department of Biological Sciences and Biotechnology, College of Life Sciences and Nanotechnology, Hannam University, Daejeon, Republic of Korea; 5 Center for Cognition and Sociality, Institute for Basic Science (IBS), Daejeon, Republic of Korea

**Keywords:** ATG4B, Atg8, autophagy, *Bombyx mori*, LC3, N-degron, protein processing, protein stability

## Abstract

Autophagy-related protein 8 (ATG8) family proteins are ubiquitin-like modifiers that undergo ATG4-mediated processing before lipid conjugation. However, the structural and functional diversification of insect ATG8 proteins remains poorly understood. Here, comparative sequence analysis identified a 248-amino-acid LC3C-related protein from the silkworm *Bombyx mori* (BmLC3) containing two tandem LC3-like regions separated by an internal glycine at position 135 (G135), in addition to the canonical C-terminal glycine at position 248 (G248). In HeLa cells, N-terminally GFP-tagged BmLC3 generated a lower-molecular-weight product, whereas substitution of G135 with alanine prevented its formation. BmLC3 processing was markedly reduced in ATG4B-knockout cells and restored by expression of *B. mori* ATG4B (BmATG4B), demonstrating ATG4B-dependent cleavage at the internal junction. An isolated GFP-tagged C-terminal BmLC3 region showed increased punctate localization following starvation and bafilomycin A1 treatment, while BmATG4B also processed membrane-tethered mammalian ATG8 reporters in a cell-based cleavage assay. In a tandem fluorescent BmLC3 reporter, cleavage generated a C-terminal product beginning with Tyr136 that showed little mRFP signal, whereas Y136M increased both mRFP fluorescence and the mRFP/GFP band-intensity ratio. Together, these findings reveal a previously unrecognized mode of ATG8 diversification and support a model in which internal proteolysis couples fragment generation to N-terminal residue-dependent control of C-terminal product stability.

## 1 Introduction

Macroautophagy (hereafter autophagy) captures cytoplasmic material in double-membrane autophagosomes and delivers it to lysosomes or vacuoles for degradation. ATG8-family ubiquitin-like proteins are central components of this pathway. ATG8 precursors are cleaved by ATG4 proteases to expose a glycine that is subsequently conjugated to phosphatidylethanolamine through the ATG7-ATG3 conjugation system. Membrane-bound ATG8 proteins contribute to autophagosome expansion, closure, cargo recruitment, and membrane dynamics (Ichimura et al., 2000; Shpilka et al., 2011; Maruyama and Noda, 2018; Nakatogawa, 2020). In mammalian cells, ATG4B is a major contributor to LC3/GABARAP processing, although other ATG4 isoforms can act in substrate- and context-dependent manners (Agrotis et al., 2019).

ATG8 proteins share a conserved ubiquitin-like fold but have diversified across eukaryotes. Mammalian ATG8 proteins are divided into LC3 and GABARAP subfamilies with overlapping and specialized functions, whereas plants contain expanded ATG8 repertoires that support development and stress adaptation (Shpilka et al., 2011; Bu et al., 2020; Varga et al., 2022, Sora et al., 2020). In insects, autophagy contributes to starvation responses, metamorphosis, and tissue remodeling, and canonical ATG8 processing and lipidation have been documented in several lepidopteran systems (Khoa and Takeda, 2012; Gai et al., 2013; Romanelli et al., 2014; Zhang et al., 2014, Zhang et al., 2009). Nevertheless, noncanonical insect ATG8 architectures and the post-cleavage fate of their products remain poorly characterized.

We identified a long LC3C-like sequence from *B. mori*, designated BmLC3, with two LC3-like regions in one open reading frame. BmLC3 contains an internal G135 followed by Tyr136 and a second glycine at G248. We therefore tested whether BmLC3 undergoes internal ATG4B-dependent processing and whether the resulting C-terminal reporter is regulated by its newly exposed Tyr136. Cell-based cleavage, localization, and tandem-reporter assays support a model in which internal processing and differential fragment stability are coupled in this unusual ATG8 protein.

## 2 Materials and methods

### 2.1 Sequence analysis and phylogenetic reconstruction

Insect ATG8 protein sequences were retrieved from the National Center for Biotechnology Information (NCBI) protein database using BLASTp searches, together with selected human and yeast reference ATG8 proteins. The full species names, sequence labels, NCBI protein accession numbers, and figure-panel usage for all sequences included in Figures 1, 2 and Supplementary Figure S1 are provided in Supplementary Table S2. Amino-acid sequences were aligned with Clustal Omega and visualized with ESPript 3.0. Neighbor-joining trees were generated in MEGA X, and branch support was evaluated using 1,000 bootstrap replicates. The analysis was intended as a comparative classification of the selected sequence set rather than an exhaustive reconstruction of ATG8 evolution.

**FIGURE 1:**
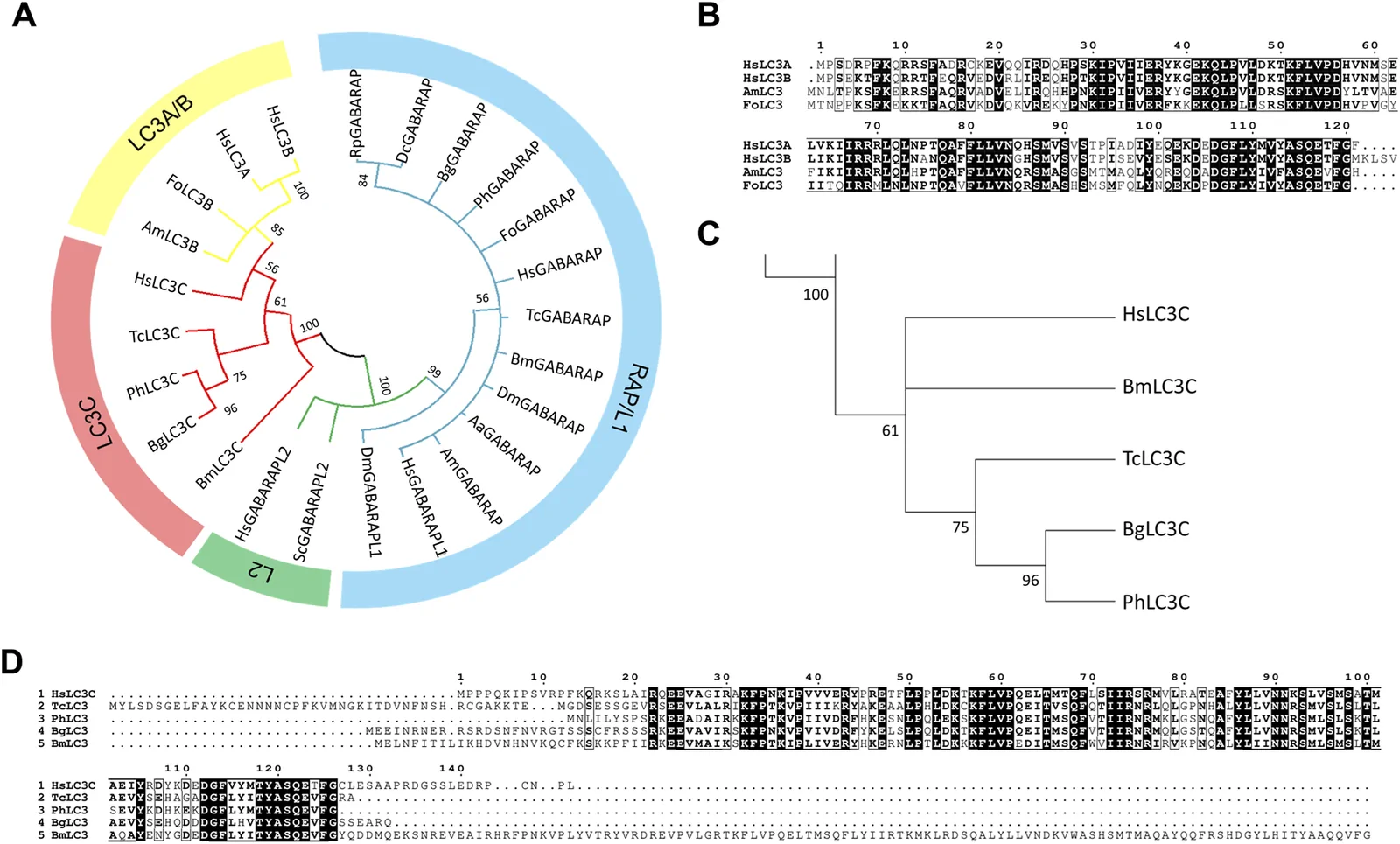
Comparative classification of selected insect ATG8 proteins places BmLC3 in an LC3C-like group **(A)** Neighbor-joining tree of selected insect, human, yeast, and other reference ATG8 proteins. Branch colors indicate LC3A/B-like, LC3C-like, GABARAP/GABARAPL1-like, and GABARAPL2-like groups; bootstrap support values are shown at selected nodes **(B)** Representative alignment of selected LC3A/B-like proteins **(C)** Enlarged LC3C-like subtree containing BmLC3 **(D)** Alignment of selected LC3C-like proteins highlighting the extended tandem-domain architecture of BmLC3. Sequences were obtained from *Homo sapiens*, *Apis mellifera*, *Fopius arisanus*, *Tribolium castaneum*, *Pediculus humanus corporis*, *Blattella germanica*, *Bombyx mori*, *Saccharomyces cerevisiae*, *Drosophila melanogaster*, *Aedes aegypti*, *Diaphorina citri*, and *Rhodnius prolixus*; full sequence labels and NCBI accessions are listed in Supplementary Table S2.

**FIGURE 2:**
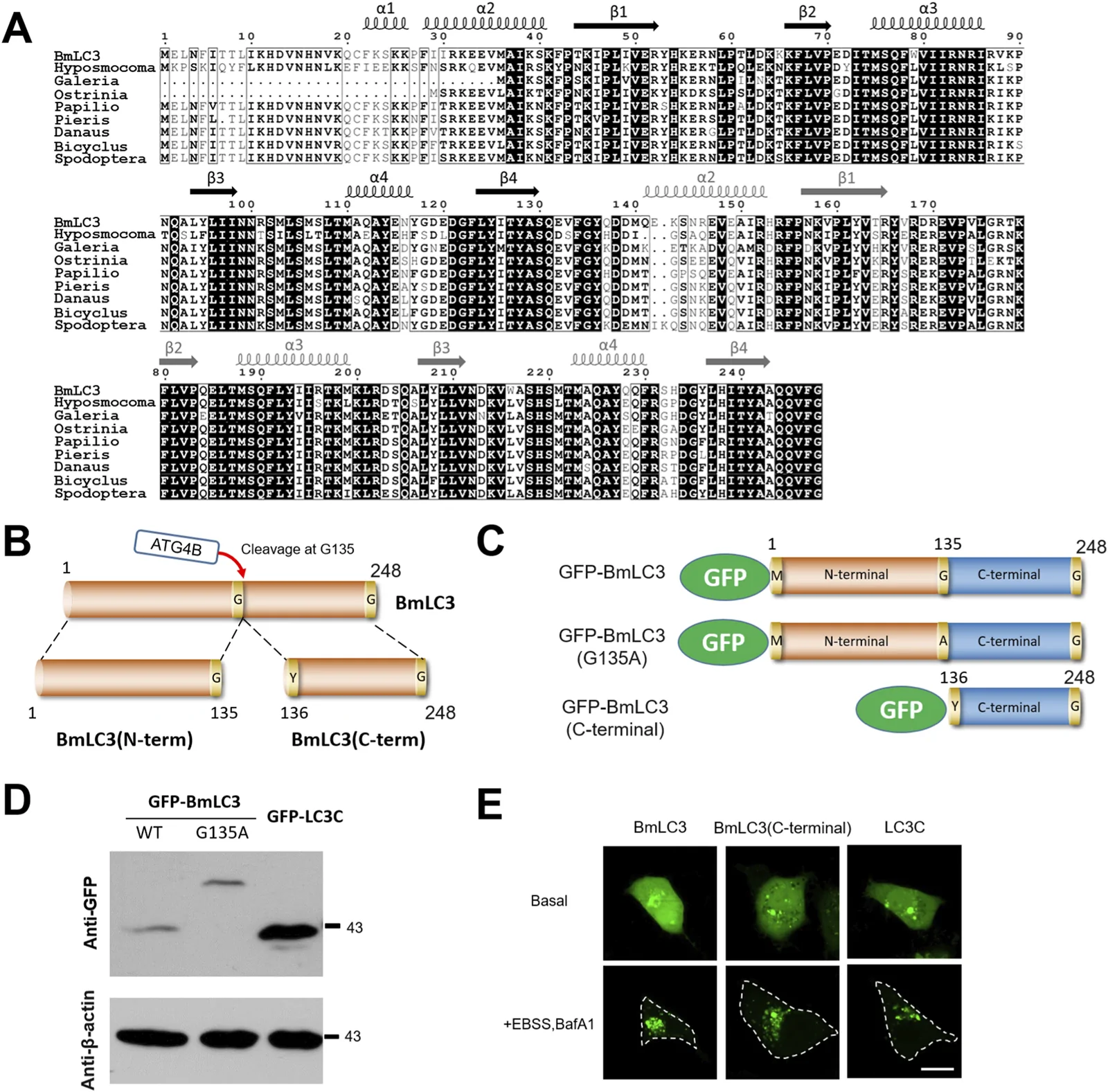
BmLC3 contains a tandem LC3-like architecture and undergoes G135-dependent processing **(A)** Alignment of BmLC3 with representative lepidopteran LC3-like sequences. The internal G135-Tyr136 junction and terminal G248 are indicated **(B)** Schematic of full-length BmLC3 and the products predicted after cleavage at G135 **(C)** GFP-tagged BmLC3 constructs used in the cell-based assays **(D)** Immunoblot showing the lower-molecular-weight wild-type GFP-BmLC3 species and the higher-molecular-weight G135A species; GFP-LC3C is shown as a size reference **(E)** Representative live-cell images of GFP-BmLC3, GFP-BmLC3(C-terminal), and GFP-LC3C under basal conditions or after 2 h of Earle’s balanced salt solution plus 200 nM bafilomycin A1. Scale bar, 10 μm.

### 2.2 DNA constructs

The BmLC3B DNA was amplified from *Bombyx mori* cDNA using PCR. For BmATG4B, the DNA was chemically synthesized based on the nucleotide sequence retrieved from the NCBI database (accession no. XP_037866680). The *B. mori* ATG4B coding sequence was inserted into C1-3xFLAG using KpnI and BamHI. EGFP-BmLC3, EGFP-BmLC3(G248A), and an EGFP-tagged C-terminal BmLC3 construct beginning at residue 136 were generated in pcDNA3.1(+)-EGFP using restriction cloning or Gibson Assembly Master Mix (New England Biolabs, M5510). Tandem reporters comprised EGFP-BmLC3(G248A)-mRFP and EGFP-BmLC3(Y136M/G248A)-mRFP; G248A was included to prevent additional processing at the terminal glycine. MyrPalm-LC3B-GFP, MyrPalm-LC3C-GFP, MyrPalm-GABARAPL1-GFP, and MyrPalm-GABARAPL2-GFP reporters were generated in a pCMV backbone. Primer sequences are provided in Supplementary Table S1.

### 2.3 Cell culture, transfection, and treatments

Wild-type HeLa cells and ATG4B-knockout HeLa cells (Abcam, ab265814) were maintained in Dulbecco’s modified Eagle medium supplemented with 10% fetal bovine serum and penicillin-streptomycin at 37 °C in 5% CO_2_. Cells were seeded in eight-well imaging chambers (ibidi, 80,828) or 10-cm culture dishes and transfected with plasmid DNA using Lipofectamine 2000 (Life Technologies) 16–24 h before analysis. To induce autophagy and inhibit lysosomal acidification, cells were incubated in Earle’s balanced salt solution containing 200 nM bafilomycin A1 for 2 h. Construct amounts were adjusted empirically to obtain comparable expression across conditions.

### 2.4 Confocal microscopy

Live-cell fluorescence images were acquired with an inverted Zeiss LSM 900 laser-scanning confocal microscope and processed using ZEN software (Carl Zeiss). GFP was excited at 488 nm and detected at 508–543 nm; mRFP was excited at 561 nm and detected at 578–649 nm. Identical acquisition settings were used for conditions compared within an experiment. Images shown are representative of at least three independent experiments.

### 2.5 Immunoblotting

Cells were lysed in 50 mM Tris-HCl (pH 7.5), 150 mM NaCl, 1% Triton X-100, 5 mM EDTA, and protease inhibitors. Equal protein amounts were separated by SDS-PAGE, transferred to polyvinylidene difluoride membranes, and incubated with primary antibodies overnight at 4 °C. Membranes were washed and incubated with horseradish peroxidase-conjugated secondary antibodies for 1 h, and signals were detected using WesternBright ECL (Advansta, K-12045-D50). Primary antibodies were anti-GFP (Santa Cruz Biotechnology, sc-9996; 1:2,000), anti-beta-actin (Santa Cruz Biotechnology, sc-47778; 1:5,000), anti-FLAG (Sigma-Aldrich, F1804; 1:1,000), and anti-ATG4B (Abcam, ab154843; 1:2,000). Anti-mouse (Santa Cruz Biotechnology, sc-2005) and anti-rabbit (sc-2313) secondary antibodies were used at 1:5,000.

### 2.6 Quantification and reproducibility

Band intensities were quantified by densitometry, and the mRFP signal was normalized to the corresponding GFP signal. Figure 3D shows five replicate measurements as individual points with the mean ± SEM. Groups were compared using a two-tailed unpaired Student’s t-test; P < 0.05 was considered statistically significant. All other experiments were performed independently at least three times, and representative microscopy and immunoblot data are shown. No data were excluded.

**FIGURE 3:**
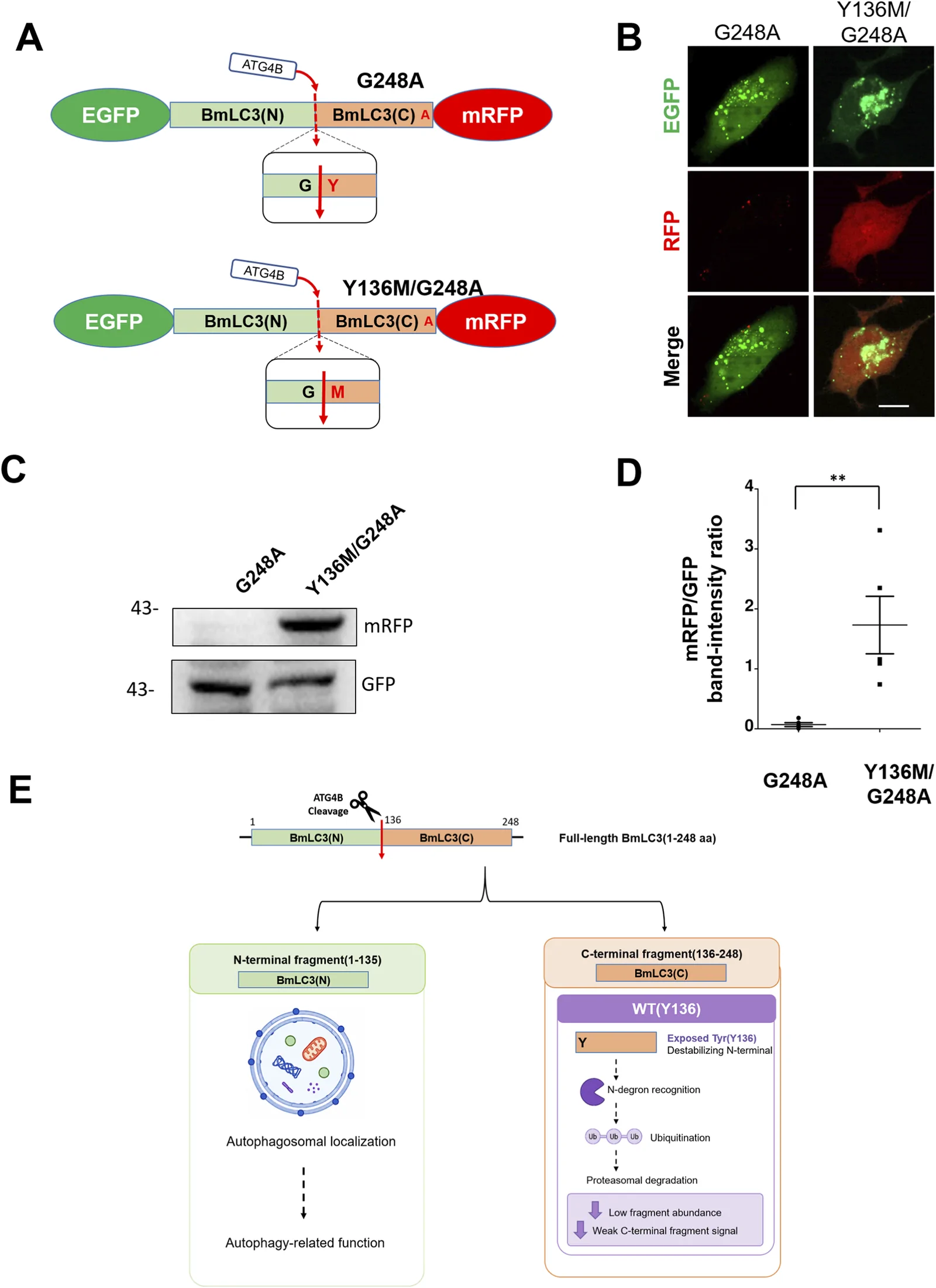
Internal cleavage exposes Tyr136 and limits the abundance of the C-terminal BmLC3 reporter **(A)** Tandem fluorescent reporters. Cleavage after G135 generates an EGFP-tagged N-terminal product and an mRFP-tagged C-terminal product beginning with Tyr136 or Met136; G248A prevents additional terminal processing **(B)** Representative fluorescence images and enlarged representative-cell views of cells expressing G248A or Y136M/G248A reporters **(C)** Immunoblot detection of mRFP- and EGFP-tagged reporter products **(D)** Densitometric quantification of the mRFP/GFP band-intensity ratio. Points represent five replicate measurements; bars show the mean ± SEM. P = 0.0086, two-tailed unpaired Student’s t-test **(E)** Proposed model: ATG4B cleavage after G135 separates the putative autophagy-related N-terminal fragment from BmLC3 **(C)**. The newly exposed Tyr136 may act as a destabilizing N-terminal residue that promotes N-degron recognition, ubiquitination, and proteasomal degradation, thereby limiting C-terminal fragment abundance. The downstream pathway in **(E)** is a testable model and was not directly validated in this study. Scale bar, 10 μm.

## 3 Results

### 3.1 Selected insect ATG8 proteins segregate into LC3- and GABARAP-related groups

A neighbor-joining analysis of selected insect ATG8 proteins and human and yeast references resolved four expected groups: LC3A/B-like, LC3C-like, GABARAP/GABARAPL1-like, and GABARAPL2-like proteins (Figure 1A). Human LC3A and LC3B associated with the LC3A/B-like group, whereas human LC3C and several insect LC3-like sequences formed an LC3C-related group (Figures 1A–C). BmLC3 clustered within this selected LC3C-like set. Sequence alignments showed conservation of the ATG8 ubiquitin-like core and highlighted the unusual extended C-terminal region of BmLC3 (Figures 1B,D). A broader alignment of GABARAP-related insect sequences is provided in Supplementary Figure S1.

### 3.2 BmLC3 contains a tandem LC3-like architecture and undergoes G135-dependent processing

BmLC3 is 248 amino acids long and contains two LC3-like regions separated at an internal glycine, G135; the second region begins with Tyr136 and ends at G248 (Figures 2A,B). Similar extended arrangements and conservation of the G135-Tyr136 junction were observed in several lepidopteran sequences included in the alignment (Figure 2A). To test whether the internal glycine was required for processing, we compared N-terminally GFP-tagged wild-type BmLC3 with G135A. Wild-type GFP-BmLC3 generated a lower-molecular-weight band close to the size of GFP-LC3C, whereas G135A accumulated as a higher-molecular-weight species (Figures 2C,D). These results indicate G135-dependent processing at or close to the internal junction.

### 3.3 The GFP-stabilized C-terminal BmLC3 region retains puncta-forming capacity

We next expressed the C-terminal region beginning at residue 136 with an N-terminal GFP tag, which masks the native Tyr136 (Figure 2E). Under basal conditions, GFP-BmLC3, GFP-BmLC3(C-terminal), and GFP-LC3C were predominantly diffuse. Starvation in the presence of bafilomycin A1 increased punctate fluorescence for all three constructs (Figure 2E). Thus, the C-terminal BmLC3 region contains information sufficient for puncta formation when stabilized by an N-terminal GFP tag. This experiment does not establish the behavior of the untagged cleavage product that begins with Tyr136.

### 3.4 BmATG4B restores BmLC3 processing and cleaves mammalian ATG8 reporters

In wild-type HeLa cells, GFP-BmLC3 was detected predominantly as a lower-molecular-weight species. ATG4B-knockout cells accumulated a higher-molecular-weight species, and expression of FLAG-BmATG4B restored the lower band (Figure 4A). Alignment of human ATG4B and BmATG4B showed conservation across the catalytic region, including the catalytic cysteine-containing segment (Figure 4B). We also used membrane-tethered MyrPalm-ATG8-GFP reporters, in which ATG4-dependent cleavage releases GFP from the plasma membrane (Figure 4C). LC3B, LC3C, GABARAPL1, and GABARAPL2 reporters were diffuse in wild-type cells but membrane-enriched in ATG4B-knockout cells. Expression of either BmATG4B or human ATG4B restored diffuse fluorescence (Figure 4D). These cell-based results show that BmATG4B can process BmLC3 and multiple mammalian ATG8 reporters.

**FIGURE 4:**
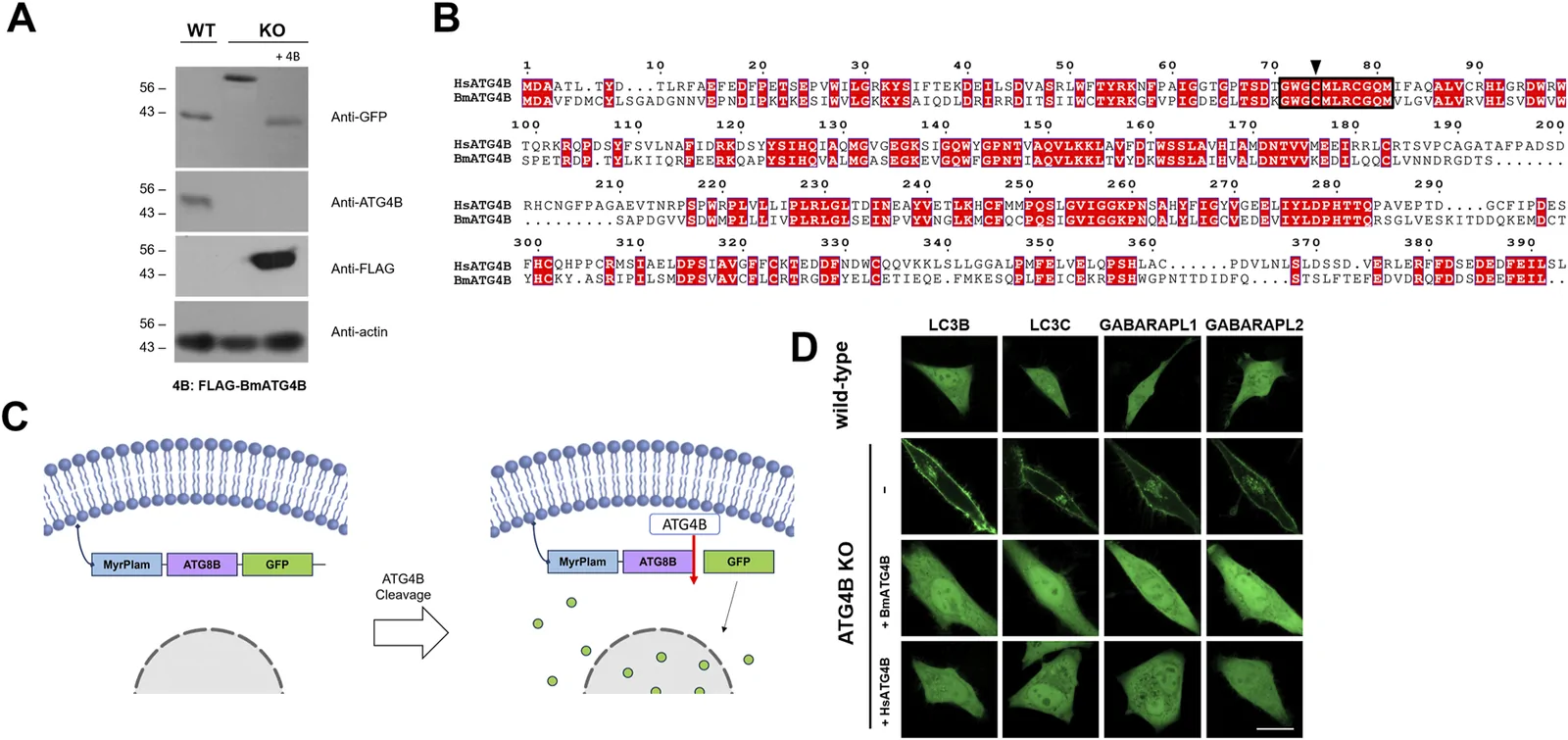
BmATG4B processes BmLC3 and mammalian ATG8 reporters in ATG4B-knockout cells **(A)** Immunoblot of GFP-BmLC3 in wild-type HeLa cells, ATG4B-knockout HeLa cells, and ATG4B-knockout cells expressing FLAG-BmATG4B **(B)** Alignment of human ATG4B and BmATG4B; the catalytic cysteine-containing region is indicated **(C)** Principle of the MyrPalm-ATG8-GFP reporter: uncleaved reporter remains membrane-associated, whereas ATG4-dependent cleavage releases GFP into the cytosol **(D)** Representative images of LC3B, LC3C, GABARAPL1, and GABARAPL2 reporters in wild-type cells, ATG4B-knockout cells, or ATG4B-knockout cells expressing BmATG4B or human ATG4B. Scale bar, 10 μm.

### 3.5 Tyr136 limits the abundance of the cleaved C-terminal BmLC3 reporter

To compare the two products generated at the internal junction, we constructed tandem reporters containing EGFP on the N-terminal BmLC3 region and mRFP on the C-terminal region (Figure 3A). G248 was replaced by alanine to prevent additional processing at the terminal glycine. After G135-dependent cleavage, the mRFP-tagged C-terminal product of the G248A reporter begins with Tyr136. This reporter produced readily detectable EGFP but little mRFP fluorescence (Figure 3B). Replacing Tyr136 with methionine restored a prominent mRFP signal. Immunoblotting likewise showed low abundance of the mRFP-tagged C-terminal product in the G248A reporter and increased abundance in the Y136M/G248A reporter (Figure 3C). Densitometric analysis confirmed that the mRFP/GFP band-intensity ratio was significantly higher for Y136M/G248A than for G248A (n = 5, P = 0.0086, two-tailed unpaired Student’s t-test; Figure 3D). Thus, Tyr136 is a determinant of the low steady-state abundance of the cleaved C-terminal reporter. These observations support the working model in Figure 3E, in which internal cleavage exposes Tyr136 as a destabilizing N-terminal residue that may promote N-degron recognition, ubiquitination, and proteasomal turnover. The proposed downstream pathway was not directly tested.

## 4 Discussion

This study identifies BmLC3 as a noncanonical, tandem-domain LC3-like ATG8 protein and provides cell-based evidence for internal processing. G135 was required for formation of the lower-molecular-weight GFP-BmLC3 species, processing was impaired in ATG4B-knockout cells, and BmATG4B restored the processed form. Together with the lepidopteran sequence alignment, these observations suggest that selected insect ATG8 proteins may have undergone greater architectural diversification than is apparent from canonical single-domain ATG8 proteins.

The designation of ATG4B as a cysteine protease refers to its catalytic nucleophile (Cys74 in human ATG4B), not to a cysteine residue at the substrate scissile bond. Structural studies show that the ATG8 tail enters the ATG4B active-site groove and that cleavage occurs after the P1 glycine, thereby exposing the glycine carboxylate required for lipid conjugation (Satoo et al., 2009; Maruyama and Noda, 2018). The G135A result is therefore consistent with recognition of internal Gly135, although direct N-terminal sequencing or mass spectrometry will be required to establish the exact cleavage bond.

BmATG4B also processed MyrPalm reporters containing human LC3B, LC3C, GABARAPL1, or GABARAPL2. Cross-species processing is consistent with conservation of the ATG4-ATG8 recognition machinery (Seo et al., 2016), although overexpression-based reporter assays do not define endogenous substrate preference or catalytic efficiency. Biochemical assays with purified proteins and measurements in insect cells will be required to compare the specificity of BmATG4B with that of mammalian ATG4 proteases.

The most distinctive consequence of internal cleavage is exposure of Tyr136 at the N terminus of the C-terminal product. The Y136M substitution increased mRFP fluorescence, immunoblot detection, and the normalized mRFP/GFP band-intensity ratio. Bulky hydrophobic N-terminal residues, including Tyr, can act as destabilizing residues in the Arg/N-degron pathway, whereas methionine is generally stabilizing (Gonda et al., 1989; Tasaki et al., 2012; Varshavsky, 2019). The results therefore support a working model in which newly exposed Tyr136 directs N-degron recognition, ubiquitination, and proteasomal turnover of C-terminal BMLC3. However, the present experiments establish Tyr136-dependent abundance control, not the downstream degradation route. Cycloheximide-chase experiments, proteasome inhibition, ubiquitination analysis, and perturbation of candidate N-recognins will be required to test this model.

Several limitations define the appropriate scope of the conclusions. The phylogeny uses a selected sequence set and should not be interpreted as a comprehensive evolutionary analysis. Endogenous BmLC3 transcript and protein expression, the exact cleavage bond, lipidation, and turnover have not been established in *B. mori* tissues or cells. Transcript-level validation is also important to exclude strain-specific annotation or assembly artifacts for the extended open reading frame. Finally, the GFP-tagged C-terminal construct masks Tyr136 and therefore reports the targeting capacity of a stabilized domain rather than the behavior of the native cleavage product. Despite these limitations, the results establish a testable framework in which one ATG4B cleavage event assigns distinct post-cleavage fates to two linked LC3-like regions: a putative autophagy-related N-terminal fragment and a Tyr136-sensitive C-terminal fragment. Although the present study is mechanistic rather than immediately translational, this lineage-specific architecture provides a comparative framework for testing whether ATG8 diversification contributes to starvation adaptation, metamorphic tissue remodeling, or host-pathogen responses in lepidopterans.

## Data Availability

The datasets presented in this study can be found in online repositories. The names of the repository/repositories and accession number(s) can be found in the article/Supplementary Material.
