# Supplementary material for: ATG4B-dependent processing of tandem-domain BmLC3 exposes a Tyr136-regulated C-terminal fragment
Source: Front Cell Dev Biol. 2026 Sep 29;14:1957836. doi: 10.3389/fcell.2026.1957836 (PMC13658554; doi:10.3389/fcell.2026.1957836)
Supplement: Supplementary file 1 [file Table1.docx]

**Supplementary Table S1. Primers used in this study**

| **Primer name** | **Primer sequence (5’-3’)** |
| --- | --- |
| BmLC3-RT-S | GCTGCGTTCAGTGTACTTGC |
| BmLC3-RT-A1 | AGCTTATCTTATAAACTACA |
| BmLC3-RT-A2 | TGTGCTTTAAAATGAATGCCATA |
| BmLC3-BamHI-S | CGCGGATCCATGGAGCTAAATTTT |
| BmLC3-NotI-A | AAGAATGCGGCCGCTCATCCGAAGACTTG |
| BmLC3(G135A)-re-A | GCATATCATCCTGGTAGGCGAACAACTCTTG |
| BmLC3(G135A)-re-S | CTACCAGGATGATATGCA |
| BmLC3(C-terminal)-BamHI-S | CGCGGATCCTACCAGGATGATATGCAG |
| BmLC3(G248A)-mRFP-ASS-A | GTCCTCGGAGGAGGCCATTGCGAAGACTTGTTGTGCTG |
| BmLC3-mRFP-ASS-S | ATGGCCTCCTCCGAGGAC |
| mRFP(stop)-XbaI-ASS-A | CGGGTTTAAACGGGCCCTCTAGACTCGAGCTCAGGCGCCGGTGGAGTGGCG |
| BmLC3(Y136M)-ASS-S | TTCCTGCATATCATCCTGCATGCCGAACACCTCTTGAGA |
| BmLC3(C-terminal, Y136M)-ASS-S | CAGGATGATATGCAGGAA |
| D3-KoM-MyrPalm-F | AGCGTTTAAACTTAAGCTTGCCACCATGGGTTGTTGTAACTCTAAGCGG |
| MyrPalm-2xGGGGS-R | CCACCGGATCCTCCTCCTCCGGTACCATCCTTCCGCTTAGAGTTACAAC |
| 2xGGGGS-LC3B-F | GGAGGAGGATCCGGTGGTGGTGGTTCTATGCCGTCGGAGAAGACC |
| 2xGGGGS-LC3C-F | GGAGGAGGATCCGGTGGTGGTGGTTCTATGCCGCCTCCACAGAAA |
| 2xGGGGS-RAP_L1-F | GGAGGAGGATCCGGTGGTGGTGGTTCTATGAAGTTCCAGTACAAG |
| 2xGGGGS-RAP_L2-F | GGAGGAGGATCCGGTGGTGGTGGTTCTATGAAGTGGATGTTCAAG |
| LC3B [G-M]-R | CATCCCGAACGTCTCCTGGGAGGC |
| LC3B [G-M]-eGFP-F (ASS) | TCCCAGGAGACGTTCGGGATGGTGAGCAAGGGCGAGGAGCTG |
| LC3C [G-M]-R | CATGCCAAATGTCTCCTGGGAGGC |
| LC3C [G-M]-eGFP-F (ASS) | TCCCAGGAGACATTTGGCATGGTGAGCAAGGGCGAGGAGCTG |
| RAP_L1 [G-M]-R | CATCCCATAGACACTCTCATCACT |
| RAP_L1 [G-M]-eGFP-F (ASS) | GATGAGAGTGTCTATGGGATGGTGAGCAAGGGCGAGGAGCTG |
| RAP_L2 [G-M]-R | CATGCCAAAAGTGTTCTCTCCGCT |
| RAP_L2 [G-M]-eGFP-F (ASS) | GGAGAGAACACTTTTGGCATGGTGAGCAAGGGCGAGGAGCTG |
| eGFP_pcDNA3.1 V-R (ASS) | GGCCCTCTAGACTCGAGCCTACTTGTACAGCTCGTCCATGCC |
