# Supplementary material for: ATG4B-dependent processing of tandem-domain BmLC3 exposes a Tyr136-regulated C-terminal fragment
Source: Front Cell Dev Biol. 2026 Sep 29;14:1957836. doi: 10.3389/fcell.2026.1957836 (PMC13658554; doi:10.3389/fcell.2026.1957836)
Supplement: Supplementary file 2 [file Table2.docx]

**Supplementary Table S2**

Protein sequences used for comparative alignment and phylogenetic classification

**Dataset:** Figures 1-2 and Supplementary Figure S1

**Database:** NCBI Protein

**Checked:** August 18, 2026

| **Figure label** | **Species** | **Protein annotation** | **NCBI accession** | **Panel(s)** |
| --- | --- | --- | --- | --- |
| HsLC3A | *Homo sapiens* | MAP1LC3A, isoform a | NP_115903.1 | Fig. 1A-B |
| HsLC3B | *Homo sapiens* | MAP1LC3B | NP_073729.1 | Fig. 1A-B |
| AmLC3B | *Apis mellifera* | MAP1LC3A-like | XP_006565988.1 | Fig. 1A-B |
| FoLC3B | *Fopius arisanus* | MAP1LC3A-like | XP_011308596.1 | Fig. 1A-B |
| HsLC3C | *Homo sapiens* | MAP1LC3C | NP_001004343.1 | Fig. 1A,C-D |
| TcLC3C | *Tribolium castaneum* | MAP1LC3C | XP_015836212.1 | Fig. 1A,C-D |
| PhLC3C | *Pediculus humanus corporis* | MAP1LC3-like | XP_002427959.1 | Fig. 1A,C-D |
| BgLC3C | *Blattella germanica* | MAP1LC3C | PSN34210.1 | Fig. 1A,C-D |
| BmLC3C / BmLC3 | *Bombyx mori* | MAP1LC3C, tandem-domain isoform | XP_004931349.1 | Fig. 1A,C-D; Fig. 2A |
| ScGABARAPL2  (tree label) | *Saccharomyces cerevisiae* | Atg8 | NP_009475.1 | Fig. 1A |
| DmGABARAPL2 | *Drosophila melanogaster* | Atg8b, isoform A | NP_650649.1 | Fig. 1A; Fig. S1 |
| HsGABARAPL2 | *Homo sapiens* | GABARAPL2 | NP_009216.1 | Fig. 1A |
| HsGABARAPL1 | *Homo sapiens* | GABARAPL1, isoform 2 | NP_113600.1 | Fig. 1A; Fig. S1 |
| HsGABARAP | *Homo sapiens* | GABARAP | NP_009209.1 | Fig. 1A; Fig. S1 |
| AmGABARAP / AmATG8 | *Apis mellifera* | GABARAP | XP_001120069.1 | Fig. 1A; Fig. S1 |
| AaGABARAP / AaATG8 | *Aedes aegypti* | GABARAP | XP_001652571.1 | Fig. 1A; Fig. S1 |
| DmGABARAP / DmATG8a | *Drosophila melanogaster* | Atg8a, isoform A | NP_727447.1 | Fig. 1A; Fig. S1 |
| BmGABARAP / BmATG8 | *Bombyx mori* | Atg8 | NP_001040244.1 | Fig. 1A; Fig. S1 |
| TcGABARAP / TcATG8 | *Tribolium castaneum* | GABARAP | XP_973073.1 | Fig. 1A; Fig. S1 |
| FoGABARAP / FoATG8 | *Fopius arisanus* | GABARAP | JAG71110.1 | Fig. 1A; Fig. S1 |
| PhGABARAP / PhATG8 | *Pediculus humanus corporis* | GABARAP | XP_002424431.1 | Fig. 1A; Fig. S1 |
| BgGABARAP / BgATG8 | *Blattella germanica* | GABARAP | PSN30272.1 | Fig. 1A; Fig. S1 |
| DcGABARAP / DcATG8 | *Diaphorina citri* | GABARAP, isoform X3 | XP_008471089.1 | Fig. 1A; Fig. S1 |
| RpGABARAP / RpATG8 | *Rhodnius prolixus* | GABARAP | JAA75906.1 | Fig. 1A; Fig. S1 |
| Hyposmocoma | *Hyposmocoma kahamanoa* | tandem LC3-like protein | XP_026332290.1 | Fig. 2A |
| Galleria | *Galleria mellonella* | LGG-2-like tandem LC3 protein | XP_026761486.2 | Fig. 2A |
| Ostrinia | *Ostrinia furnacalis* | tandem LC3-like protein | XP_028169585.1 | Fig. 2A |
| Papilio | *Papilio xuthus* | tandem LC3-like protein, isoform X2 | XP_013166361.1 | Fig. 2A |
| Pieris | *Pieris rapae* | tandem LC3-like protein, isoform X1 | XP_022114780.1 | Fig. 2A |
| Danaus | *Danaus plexippus* | tandem LC3-like protein, isoform X2 | XP_032522669.1 | Fig. 2A |
| Bicyclus | *Bicyclus anynana* | tandem LC3-like protein | XP_023940226.1 | Fig. 2A |
| Spodoptera | *Spodoptera litura* | Atg8-like tandem LC3 protein | XP_022815665.1 | Fig. 2A |
